# The burden of disease in seronegative myasthenia gravis: a patient-centered perspective

**DOI:** 10.3389/fimmu.2025.1555075

**Published:** 2025-04-08

**Authors:** Sophie Lehnerer, Regina Stegherr, Ulrike Grittner, Maike Stein, Lea Gerischer, Frauke Stascheit, Meret Herdick, Paolo Doksani, Andreas Meisel, Sarah Hoffmann

**Affiliations:** ^1^ Department of Neurology with Experimental Neurology, Freie Universität Berlin and Humboldt-Universität zu Berlin, Charité – Universitätsmedizin Berlin, Berlin, Germany; ^2^ Department of Neurology with Experimental Neurology, Neuroscience Clinical Research Center, Freie Universität Berlin and Humboldt-Universität zu Berlin, Charité – Universitätsmedizin Berlin, Berlin, Germany; ^3^ Center for Stroke Research Berlin, Charité – Universitätsmedizin Berlin, Berlin, Germany; ^4^ Digital Health Center, Berlin Institute of Health at Charité – Universitätsmedizin Berlin, Berlin, Germany; ^5^ Institute of Biometry and Clinical Epidemiology, Freie Universität Berlin and Humboldt-Universität zu Berlin, Charité – Universitätsmedizin Berlin, Berlin, Germany; ^6^ Department of Neurology, Beth Israel Deaconess Medical Center/Harvard Medical, School, Boston, MA, United States

**Keywords:** quality of life, antibodies, disease severity, gender, diagnostic challenge, fatigue

## Abstract

**Objective:**

Myasthenia gravis (MG) is an autoimmune disorder primarily caused by autoantibodies against the acetylcholine receptor (AChR). Approximately 15% of MG patients, categorized as seronegative (snMG), lack detectable antibodies. Due to the snMG status, there may be a diagnostic delay. Moreover, there are limited data on treatment response in comparison to AChR-Ab+ patients. This study examines the burden of disease, treatment response, and quality of life of snMG patients in comparison to AChR-ab+ MG patients and healthy controls.

**Methods:**

A questionnaire-based survey was conducted collecting sociodemographic and clinical data including antibody status, therapy, treatment response, and self-rated disease severity along with standardized assessments such as MG-ADL (activities of daily living) and the Short Form Health (SF-36, generic Health-Related Quality of Life, HRQoL). HRQoL was evaluated through matched-pairs analyses. Participants from a general health survey served as the control group. Negative binomial regression was applied to evaluate the impact of antibody status on MG-ADL.

**Results:**

Compared to AChR-ab+ patients, snMG patients (*n* = 237) were younger at symptom onset [median age 42 (IQR 30.5/53) vs. 51 (31/64) years, *p* < 0.001] and had longer diagnostic delays. Complete stable remission was less frequent in snMG patients (15.9% vs. 27.8%, *p* < 0.001), and they reported higher disease severity (52.8% medium, 9.5% severe vs. 41.9% medium, 8.5% severe, *p* = 0.005). snMG patients had higher MG-ADL scores [median 5 (IQR 2/9) vs. 3 (1/6), *p* < 0.001] and more employment restrictions (64.4% vs. 49.3%, *p* < 0.001). Furthermore, compared to healthy controls, snMG patients showed worse outcomes in all domains of the SF-36.

**Conclusion:**

The burden of disease in snMG patients is higher compared to AChR-ab+ MG due to delay in diagnosis, worse treatment response, and sociodemographic factors. These findings highlight the challenges patients and treating physicians face in snMG. There is a high need for earlier diagnosis, improved diagnostic tools, and inclusion of snMG patients in clinical trials to address their unique therapeutic challenges.

**Clinical Trial Registration:**

clinicaltrials.gov, identifier NCT03979521. Registered 7 June 2019 (retrospectively registered).

## Background

Myasthenia gravis (MG) stands as the predominant neuromuscular junction disorder mediated by autoantibodies (ab) targeting postsynaptic antigens of the neuromuscular junction. The cardinal syndrome of MG is fatigable muscle weakness improved by periods of rest. This weakness can affect a wide range of skeletal muscles, including ocular, bulbar, limb, and respiratory muscles, and potentially lead to a life-threatening crisis. The most prevalent autoantibody in MG targets the acetylcholine receptor (AChR, 75%–85%, great variability in prevalence rates across cohorts and assay methods used) (1–6). Other identified antibodies include those against muscle-specific tyrosine kinase (MuSK, 3%) and lipoprotein-related protein 4 (LRP4, present in 1%–2% of all MG cases) (7). Approximately 15% of MG patients exhibit no detectable serum autoantibodies using assays currently available in clinical routine (seronegative, snMG), making them the second largest patient subgroup after AChR-antibody-positive (AChR-ab+) MG patients (2, 3, 8). Seronegativity poses a diagnostic challenge in light of an ever-increasing reliance on laboratory diagnostics and has been shown to negatively impact timely diagnosis (9). Furthermore, snMG patients are largely underrepresented in medical research including interventional trials (10). Consequently, therapeutic monoclonal antibody treatment has emerged in the MG landscape but remains confined to AChR-ab+ and MuSK-ab+ MG patients in Europe and the United States (11–15). Solely in Japan, the FcRn blocker efgartigimod is approved for the treatment of MG regardless of antibody status, making it the only country where it is indicated for patients with snMG.

Patients with snMG often display clinical features similar to those with seropositive MG, yet they can differ significantly in terms of disease manifestation and treatment response. Recent studies have identified neurophysiological and clinical differences between snMG and AChR-ab+ MG (16). However, there is a significant gap in the literature regarding how these differences translate into patient-reported outcomes, such as quality of life, and in which specific life domains these patients may experience greater functional impairment.

In a previous study, we demonstrated that the quality of life (QoL) of overall MG patients is markedly lower compared to the general population (17). However, little is known about the disease burden across different autoantibody subgroups. Clinical experience indicates that snMG patients are often perceived as particularly challenging, with their symptoms sometimes being misinterpreted as psychosomatic, leading to diagnostic delays or even questioning of the diagnosis itself and thereby fostering a hesitancy for consequent immunosuppressive treatment.

The aim of this study was to characterize in-depth the burden of disease in snMG in comparison to AChR-ab+ MG analyzing clinical characteristics, treatment response, QoL, mental health, and sociodemographic impact like ability to work.

## Methods

### Data collection

In May 2019, 3,262 members of the German Myasthenia Gravis Society (Deutsche Myasthenie Gesellschaft, DMG) received study information and a questionnaire as well as a prestamped envelope addressed to the coordinating study center (Neuroscience Clinical Research Center, Charité - Universitätsmedizin Berlin). Patients were instructed to return the completed questionnaire without any further identifying information to ensure anonymity. No financial compensation was given. Returned questionnaires were accepted until the cutoff date—31 July 2019.

### Questionnaire

The questionnaire contained various data, in detail described in our former publication (Lehnerer et al., 2021). Stratified by autoantibody status, the following variables were assessed: gender, current age, age at symptom onset, age at diagnosis, disease severity (self-rated in mild, medium, and severe regarding the question “How severe is your MG?”), current medication, current status of therapy response (no symptoms and no medication for more than 1 year, no symptoms under medication, improved symptoms under medication, unchanged symptoms despite medication, worsened symptoms despite medication, myasthenic crisis despite medication), side effects under drug therapy (yes/no), care level [no care level, no care level but request sent to authorities, care level 1–5 (18)], the influence of one’s MG disease on family planning (yes/no), and restriction of employment (none, restriction without further specification, reduction of working hours, repeated and frequent incapacity for work, unemployment, disability, occupational disability).

All questions used for this subanalysis were asked with a checkbox option, always specified to be answered as a single or multiple-choice option. The questionnaires were scanned and processed with the software TeleForm (OpenText), version 10.9.1.

### Definitions

*Seronegative patients* are determined as patients who have self-assessed their antibody status as “No detection of antibodies” and did not select any antibody (AChR-ab, MuSK-ab, LRP4-ab) or selected “I don’t know” in the multiple-answer option. *Unknown antibody status* describes all patients who selected “I don’t know” when questioned about their antibody status. Descriptive variables are presented across all ab-subgroups. To assess the specifics of the burden of disease in snMG patients, AChR-ab-positive patients served as the comparison group.

*Complete stable remission* was defined as a status of “no symptoms and no medication for more than 1 year” and *pharmacological remission* was defined as a status of “no symptoms under medication.” *Early-onset myasthenia gravis* (*EOMG*) was defined as symptom onset before the age of 50 years, whereas *late*-*onset myasthenia gravis* (*LOMG*) was defined as symptom onset at the age of 50 years or later. *Disease duration* was defined as current age minus age at diagnosis.

### Standardized scores

To further assess the burden of disease, standardized scores in the German language version were integrated into the questionnaire and used in this subanalysis: MG-QoL15 (Myasthenia Gravis Quality of Life, i.e., MG-specific health-related Quality of Life) (19), MG-ADL (Myasthenia Gravis Activities of Daily Living Profile) (20), ESSI-D (ENRICHD Social Support Inventory) (21, 22), and HADS (Hospital Anxiety and Depression Scale) (23). In the ESSI-D (5–25-point scale), a higher score indicates better social support. In MG-QoL15 (0–60-point scale), MG-ADL (0–24-point scale), and HADS (0–21-point scale for each subscale of anxiety and depression), higher scores indicate a more severe affection. The SF-36 (Short Form Health) was used to investigate the general health-related quality of life for comparison with the general population. Subscale scores of the SF-36 were calculated while imputing missing values by the mean of the existing values if at least 50% of the existing items of the same subscale were answered (24). Fatigue was assessed using the CFQ11 containing 11 questions on physical and mental fatigue (Chalder Fatigue Scale) (25, 26). Two scoring systems, Likert scoring (0–1–2–3, total score 0–33) and bimodal scoring (0–0–1–1, total score 0–11), are commonly employed, with the latter allowing for categorization of fatigue caseness based on a cutoff score of 4 points or more. Bimodal scoring was used to calculate fatigue prevalence and the Likert scoring was used to assess fatigue severity. The current status of MG regarding therapy response was surveyed based on the MGFA-PIS to evaluate treatment outcomes and monitor disease progression (27).

### Statistical analysis

The statistical calculations were performed using IBM SPSS Statistics for Windows, Version 25.0 (Released 2017. IBM Corp., Armonk, NY) and R (version 3.5.3) software (28).

Depending on the scale and distribution of the outcome variables, appropriate descriptive statistics (mean, standard deviation, median, interquartile range, absolute and relative frequencies) are presented for all antibody constellations. Furthermore, the chi-square test and the Mann–Whitney *U*-test were used to test for group differences between AChR-ab+ and snMG patients. A two-sided significance level of *α* = 0.05 was used. No adjustment for multiple testing was applied in this exploratory study. In addition to *p*-values, standardized mean difference (SMD) is reported to describe the magnitude of the difference. An SMD smaller than 0.2 corresponds to no effect, an SMD between 0.2 and 0.5 to a small effect, an SMD between 0.5 and 0.8 to a medium effect, and an SMD greater than 0.8 to a large effect (29).

To compare restrictions in employment between snMG and AChR-ab+ patients, a multivariable logistic regression was conducted which was adjusted for gender, age, early vs. late onset, and the sum of the HADS depression and HADS anxiety scores. Results are reported as odds ratios with 95% confidence intervals and *p*-values.

To investigate how MG-ADL is impacted by antibody status (snMG vs. AChR-ab+), negative binomial regression was conducted due to the skewed distribution of the MG-ADL with a point mass of 0. The negative binomial regression was further adjusted for gender, age, early vs. late symptom onset, and the HADS depression score. Results are reported as incident rate ratio, which can be interpreted as fold change, with corresponding 95% confidence intervals and *p*-values. The reference patient is defined as a female patient, AChR-ab+, and <50 years of current age, with EOMG and HADS depression score of 4.9 (mean HADS score in the overall cohort).

To compare the general health-related quality of life of snMG to AChR-ab+ patients as well as the general population (control group), an exact sex and age group (18–49, 50–59, 60–69, 70+ years) matching was performed in a ratio of 1:2. Data for the general population came from the German Health Interview and Examination Survey for Adults (DEGS1, 2008–2011, a German-wide representative study conducted by the Robert-Koch Institute) (30). The snMG patients were matched to the AChR-ab+ patients and the general population using exact matching by gender and age groups. The subscales of the SF-36 are compared using mean, standard deviation, and SMD.

Four patients who reported an implausibly high dose of methotrexate according to clinical practice (i.e., ≥25 mg) were excluded from the mean dose calculation and categorized as missings.

### Standard protocol approvals, registrations, and patient consents

No written informed consent was obtained since data collection was completely anonymous. This project was approved by the Institutional Ethics Committee of Charité - Universitätsmedizin Berlin (reference EA1/008/19). The study was conducted in accordance to the Declaration of Helsinki and the STROBE reporting guidelines and was registered on clinicaltrials.gov (NCT03979521).

### Data availability

Data not provided in the article because of space limitations may be shared (anonymized) at the request of any qualified investigator for purposes of replicating the procedures and results.

## Results

Out of 3,262 contacted members of the DMG, 103 persons were excluded retrospectively from the response analysis, because they did not meet the inclusion criteria (i.e., congenital myasthenic syndrome or diagnosis of Lambert–Eaton myasthenic syndrome). The overall response rate was 52.5% (*n* = 1,660). Detailed patient characteristics of the study cohort have been published previously (17).

### Patient characteristics

Of all patients, 14.3% (*n* = 237) stated to be seronegative, 47.5% (*n* = 789) to be AChR-ab+, 2.2% (*n* = 36) to be MuSK-ab+, and 0.4% (*n* = 7) to be LRP4-ab+. A total of *n* = 591 patients (35.6%) selected “I don’t know” when questioned about their antibody status. **Table 1** provides an overview of patient characteristics across the antibody subgroups including unknown ab-status. Compared to AChR-ab+ patients, snMG patients were more likely to be women (69.9% women vs. 55.8%) and younger at symptom onset [median 42 (IQR 30.5/53) vs. 51 (31/64), SMD 0.33, *p* < 0.001] and experienced a longer time from symptom onset to diagnosis [median age at diagnosis 47 (IQR 36/57) vs. 53 (34/64), SMD 0.22, *p* = 0.001]. Disease duration at the time of study participation did not differ [median 9.5 (IQR 5/18) vs. 11 (5/20), SMD 0.1, *p* = 0.25] (**Table 1**).

**Table 1:** Patient demographics by antibody constellations: in each case, n (%) or median (IQR, interquartile range), SMD (standardized mean difference), p-value of the χ^2^ test (gender, disease severity), and Mann–Whitney U test are given to compare seronegative with AChR-ab+ patients.

	Missings	Total	Seronegative (SN)	AChR-ab+	SMD, *p*-value SN vs. AChR-ab+	MuSK-ab	LRP4-ab	Unknown antibody status
*n* (%)		1,660 (100)	237 (14.3)	789 (47.5)		36 (2.2)	7 (0.4)	591 (35.6)
Men, *n* (%)	4	725 (43.8)	71 (30.1)	348 (44.2)	0.29 <0.001	15 (41.7)	2 (28.6)	289 (49.1)
Women, *n* (%)		931 (56.2)	165 (69.9)	440 (55.8)		21 (58.3)	5 (71.4)	300 (50.9)
Age (years), median (IQR)	11	67 (55/77)	57.5 (49/68.2)	65 (54/75)	0.34 <0.001	62.5 (55/79)	54 (42/73.5)	74 (64/80)
Disease duration (years), median (IQR)	45	10 (5/20)	9.5 (5/18)	11 (5/20)	0.1 0.25	6.5 (5/14)	3 (1.5/9)	10 (5/20)
Age at symptom onset (years), median (IQR)	123	53 (33/65)	42 (30.5/53)	51 (31/64)	0.33 <0.001	47 (34.5/63.2)	40 (27.5/63.5)	59 (42/70)
Age at diagnosis, median (IQR)	34	55 (38/66)	47 (36/57)	53 (34/64)	0.22 0.001	54 (41/64)	50 (39/64)	61 (45/70)
Disease severity, *n*	54	1,606	231	774		35	7	559
Mild, *n* (%)		733 (45.6)	87 (37.7)	385 (49.7)	0.25 0.005	10 (28.6)	2 (28.6)	249 (44.5)
Medium, *n* (%)		728 (45.3)	122 (52.8)	324 (41.9)		16 (45.7)	4 (57.1)	262 (46.9)
Severe, *n* (%)		145 (9.0)	22 (9.5)	65 (8.4)		9 (25.7)	7 (14.3)	48 (8.6)

### Current medication and therapy response

There were no notable differences in current medication between snMG and AChR+ patients, except for higher rates of medication with pyridostigmine sustained release (50.5% vs. 37.1%, SMD 0.27, *p* = 0.001) and lower rates of medication with azathioprine (34% vs. 49.8%, SMD 0.32, *p* < 0.001) in snMG patients (**Table 2**). Complete stable remission was less frequent in snMG patients compared to AChR-ab+ MG patients (15.9% vs. 27.8%), and more snMG patients experienced unchanged (15.9% vs. 11.1%) or worsened symptoms (8.6% vs. 3.2%) (SMD 0.38, *p* < 0.001) (**Table 3**).

**Table 2:** Treatment in seronegative and AChR-ab+ patients; missing values on medication rates were present in 22 (steroids), respectively, and 20 (all other) of the snMG patients and in 66–69 of the AChR-ab+ patients (67 in steroids, 69 in methotrexate, 66 in the other medications); SMD is referring to n (%) SN vs. AChR-ab+.

Current medication	Seronegative (snMG)			AChR-ab+			
	*n* (%)	Dosage (mg) Mean (STD)	Missing dosage	n (%)	Dosage (mg) Mean (STD)	Missing dosage	SMD, *p*-value SN vs. AChR-ab+
Pyridostigmine	163 (76.9)	227 (315)	7	498 (70.4)	185 (312)	13	0.15, 0.081
Pyridostigmine sustained release	107 (50.5)	218 (146)	0	262 (37.1)	191 (130)	2	0.27, 0.001
Mycophenolate mofetil	27 (12.7)	1,200 (786)	0	104 (14.7)	1,420 (657)	2	0.06, 0.542
Steroids	59 (27.8)	9.3 (12.5)	2	180 (25.5)	10.1 (11.1)	2	0.06, 0.507
Azathioprine	72 (34)	121 (62.7)	2	352 (49.8)	115 (69.4)	5	0.32, <0.001
Methotrexate	11 (5.2)	12.5 (3.31)	2	29 (4.1)	10.3 (4.9)	3	0.05, 0.634
Cyclosporine A	1 (0.5)	100 (–)	0	6 (0.8)	200 (117)	1	0.05, 0.918

Medication current or past	n (%)	*n* (%) in the last 6 months	Missing last exposure	*n* (%)	*n* (%) in the last 6 months	Missing last exposure	SMD, *p*-value SN vs. AChR-ab+
Rituximab	14 (6.6)	9 (64.3)	1	43 (6.1)	26 (61.9)	0	0.02, 0.909
Eculizumab	0 (0.0)	–	–	6 (0.8)	5 (83.3)	0	0.13, 0.390
IVIG	43 (20.3)	23 (63.9)	7	120 (17.0)	44 (41.9)	15	0.09, 0.315
Plasmapheresis/IA	21 (9.9)	6 (28.6)	0	54 (7.6)	6 (11.3)	1	0.08, 0.360

**Table 3:** Current status of myasthenia gravis (MG) regarding treatment response: n (%), missing n = 21, SMD seronegative vs. AChR-ab+: 0.38, p-value of the χ^2^ test <0.001 and side effects under drug therapy: n (%), missing n = 24, SMD seronegative vs. AChR-ab+: 0.12, p-value of the *χ* ^2^ test 0.149 (patient without MG medication excluded from the analysis).

*n* (%)	Total	Seronegative	AChR-ab+	MuSK-ab	LRP4-ab	Unknown antibody status
Current status of MG, *n*	1,616	232	773	36	7	568
No symptoms and no medication for more than one year	135 (8.4)	37 (15.9)	215 (27.8)	2 (5.6)	1 (14.3)	48 (8.5)
No symptoms under medication	396 (24.5)	112 (48.3)	370 (47.9)	24 (66.7)	2 (28.6)	141 (24.8)
Improved symptoms under medication	773 (47.8)	6 (2.6)	11 (1.4)	2 (5.6)	1 (14.3)	265 (46.7)
Unchanged symptoms despite medication	208 (12.9)	37 (15.9)	86 (11.1)	4 (11.1)	2 (28.6)	79 (13.9)
Worsened symptoms worsen despite medication	78 (4.8)	20 (8.6)	25 (3.2)	3 (8.3)	1 (14.3)	29 (5.1)
Myasthenic crisis despite medication	26 (1.6)	20 (8.6)	66 (8.5)	1 (2.8)	0 (0.0)	6 (1.1)
Side effects, *n*	1,485	210	706	36	7	523
**Yes,** *n* (%)	714 (48.2)	114 (54.3)	341 (48.3)	17 (47.2)	4 (57.1)	238 (45.5)

### Disease severity and patient-reported outcome measures

**Figure 1:**
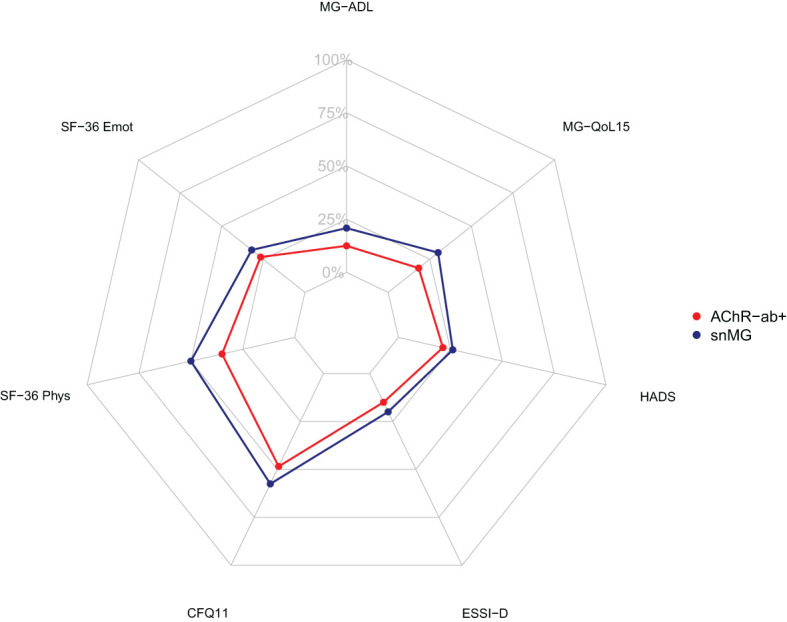
Net diagram showing higher overall burden of disease in seronegative patients (blue) compared to AChR-ab+ patients (red). The further out the lines are in the net, the higher and worse the single score value. Data integrated from the Myasthenia Gravis Activities of Daily Living Score (MG-ADL), the Myasthenia Gravis Quality of Life Score (MG-QoL15), the Hospital Anxiety and Depression Scale (HADS), the ENRICHD Social Support Inventory (ESSI-D), the Chalder Fatigue Scale (CFQ11), and the Physical Functioning (SF-36 Phys) and Emotional Wellbeing (SF-36 Emot) domain of the Short Form 36 (SF-36).

More snMG patients rated their disease severity as *medium* (52.8% vs. 41.9% in AChR-ab+) and *severe* (9.5% vs. 8.4% in AChR-ab+) (SMD 0.25, *p* = 0.005) (**Table 1**). This was also reflected in higher MG-ADL scores [median 5 (IQR 2/9) vs. 3 (1/6), SMD 0.55, *p* < 0.001] (**Table 4**). snMG patients had a higher fatigue prevalence (74.8% vs. 63.3%, SMD 0.25, *p* = 0.002) and reported higher fatigue severity than AChR-ab+ patients [CFQ11: median 19 (IQR 14/23) vs. 16 (12/21), SMD 0.33, *p* < 0.001]. Moreover, differences were observed in MG-specific quality of life [MG-QoL15: median 18 (IQR 7/30) vs. 11 (3/23), SMD 0.39, *p* < 0.001] (**Table 4**) and generic quality of life score [SF-36: median 52.9 (IQR 38/66.6) vs. 61 (44.4/75.5), SMD 0.29, *p* < 0.001] (data not shown in the table). In the HADS subdomains anxiety and depression, the difference between the snMG and AChR-ab+ patients was small (SMD 0.2, *p* = 0.006 and SMD 0.14, *p* = 0.018, respectively) (**Table 4**). Overall, snMG patients had a higher overall burden of disease as reflected in the outcome measures across various disease domains (**Figure 1**).

**Table 4:** Patient*-*reported outcome measures (PROMs) divided into different antibody constellations.

	Missings	Total	Seronegative (SN)	AChR-ab+	SMD, *p*-value SN vs. AChR-ab+	MuSK-ab	LRP4-ab	Unknown antibody status
MG-ADL, median (IQR)	87	4 (1/6)	5 (2/9)	3 (1/6)	0.55 <0.001	4.5 (3/7)	7 (5/7.5)	4 (2/6)
CFQ11 sum (Likert, fatigue severity), median (IQR)	117	17 (12/21)	19 (14/23)	16 (12/21)	0.33 <0.001	19 (14/22)	17 (12/22)	17 (12/22)
CFQ ≥ 4 (binary, fatigue prevalence), *n* (%)	117	989 (66.7)	160 (74.8)	450 (63.3)	0.25 0.002	24 (70.6)	2 (50.0)	353 (67.9)
MG-QoL15, median (IQR)	447	12 (4/25)	18 (7/30)	11 (3/23)	0.39 <0.001	17.5 (10.5/27)	31 (24.8/33.2)	12 (4/24)
HADS anxiety, median (IQR)	60	5 (3/9)	6 (3/9)	5 (3/8)	0.2 0.006	5 (2.2/7)	6 (2.5/9)	6 (3/10)
HADS depression, median (IQR)	60	5 (2/8)	5 (2/8)	4 (2/7)	0.14 0.018	4 (3/7)	7 (3.5/9)	6 (2/9)
ESSI-D, median (IQR)	151	22 (19/25)	21 (18/24)	22 (19/25)	0.21 0.009	23 (20/25)	20 (18.5/24.5)	22 (19/25)

In each case, median (IQR, interquartile range), SMD (standardized mean difference), *p*-value of the *χ* ^2^ test (gender), and Mann–Whitney *U* test are given to compare seronegative with AChR-ab+ patients.

The Pearson correlation coefficient showed moderate correlations between depression (HADS) and fatigue (CFQ11; *R* = 0.58) and quality of life (MG-QoL15r; *R* = 0.66).

**Figure 2:**
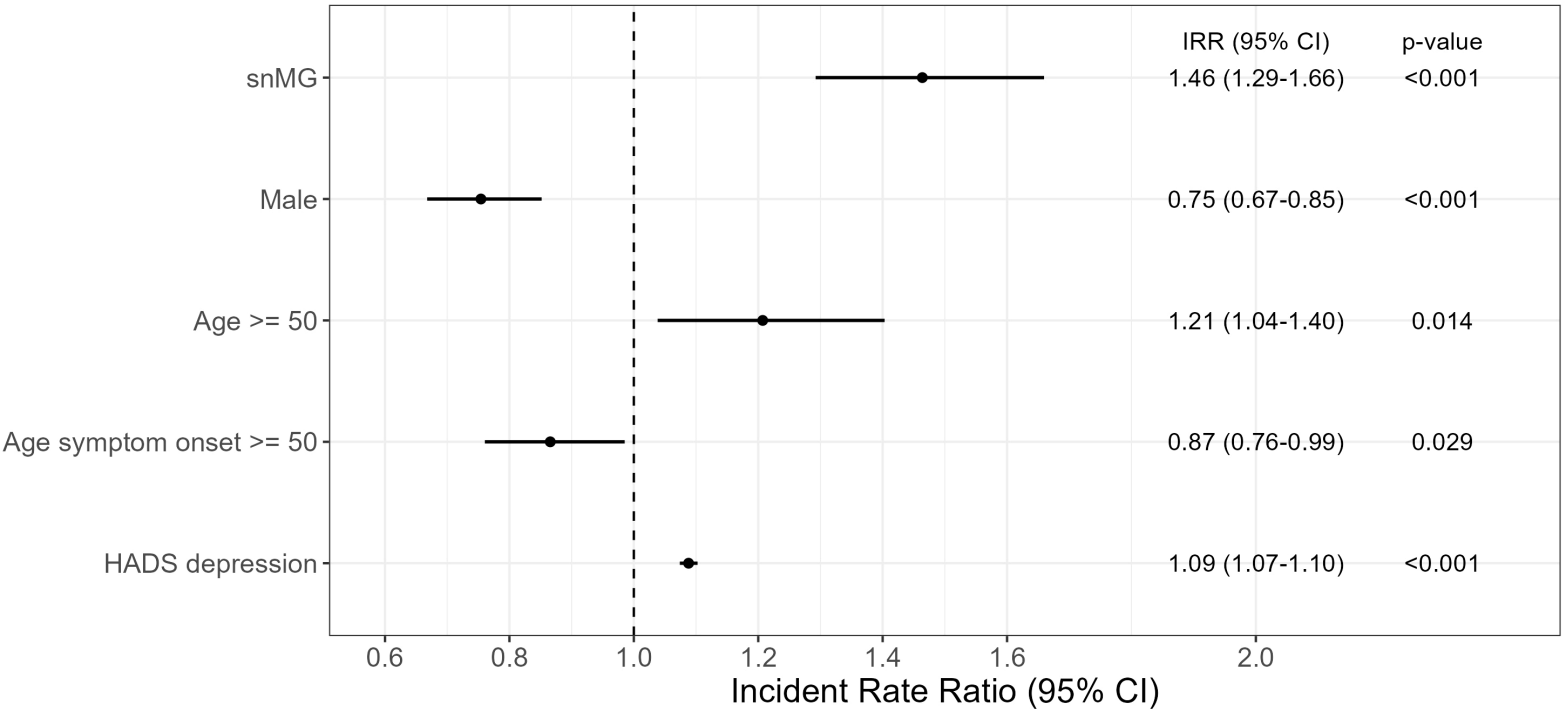
Negative binomial regression of MG-ADL. The reference patient is a woman, AChR-ab+, and <50 years of age, with EOMG and HADS depression score of 4.9 (mean HADS score in the overall cohort) and results with a mean MG-ADL of 3.57 (CI 3.14–4.06). The exp (regression coefficient) is shown, which can then be interpreted multiplicatively: e.g., snMG has 1.46 times higher MG-ADL than the reference patient (=5.21). Complete case analysis with 131 missings. CI, confidence interval.

To evaluate the effect of the antibody constellation on the MG-ADL, a negative binomial regression was performed: as reference, for an AChR-ab+, female patient, younger than 50 years of current age, with EOMG and a HADS depression score of 4.9 (mean HADS score of overall cohort), the expected mean MG-ADL is 3.57 (95% CI 3.14-4.06). In comparison, a seronegative female patient, younger than 50 years of current age, with EOMG and HADS depression score of 4.9 has an expected mean MG-ADL of 5.21 (95% CI 4.60–5.92), which is 1.46-fold (95% CI 1.29–1.66) higher than the abovementioned reference (**Figure 2**). Compared to female patients, male patients have a 0.75-fold (95% CI 0.67–0.85) lower mean MG-ADL [= 2.68 (95% CI 2.40–3.19)], given that all the other parameters remain unchanged, i.e., AChR-ab+. An age greater than 50 years at the time of study inclusion was associated with a 1.21-fold (95% CI 1.04–1.40) increase in MG-ADL, i.e., 4.32 (95% CI 3.71–5.00). In contrast, LOMG decreases the MG-ADL to 4.32 (95% CI 3.11–3.53). Furthermore, a HADS depression score higher than 4.9 slightly increases the MG-ADL to 3.89 (95% CI 3.82–3.93).

### Societal and occupational dimensions

**Figure 3:**
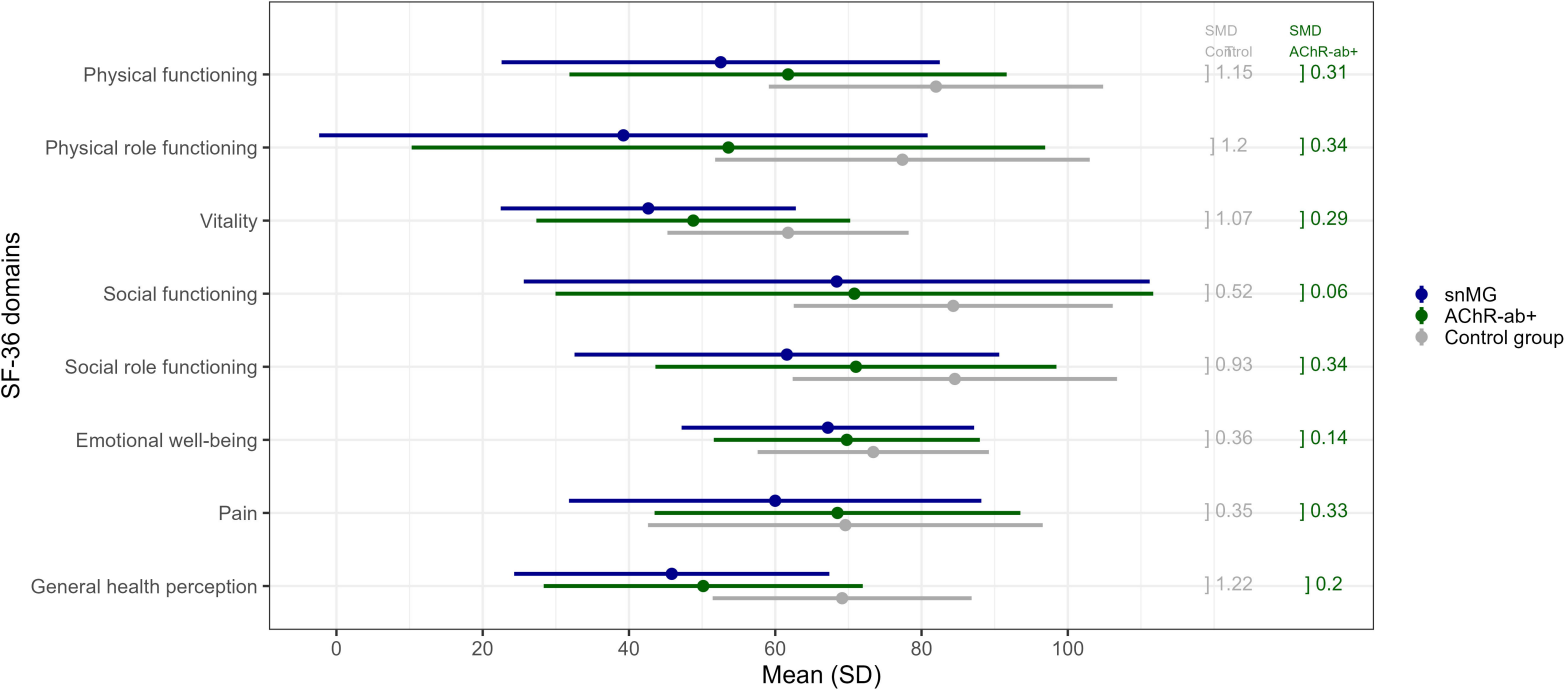
SF-36 score comparison to *the* control group: *m*ean values (and standard deviation, SD) of snMG patients (blue), AChR-ab+ MG patients (green), and the control group (general population) (gray) (raw data/values, see **Supplementary Table 1**). Standardized mean differences (SMD) between the snMG and control groups (left column, gray) and between the snMG and AChR-ab+ (right column, green) indicate a high effect if >0.8, a medium effect if 0.5–0.8, a low effect if 0.2–0.5, and no effect if <0.2.

**Figure 4:**
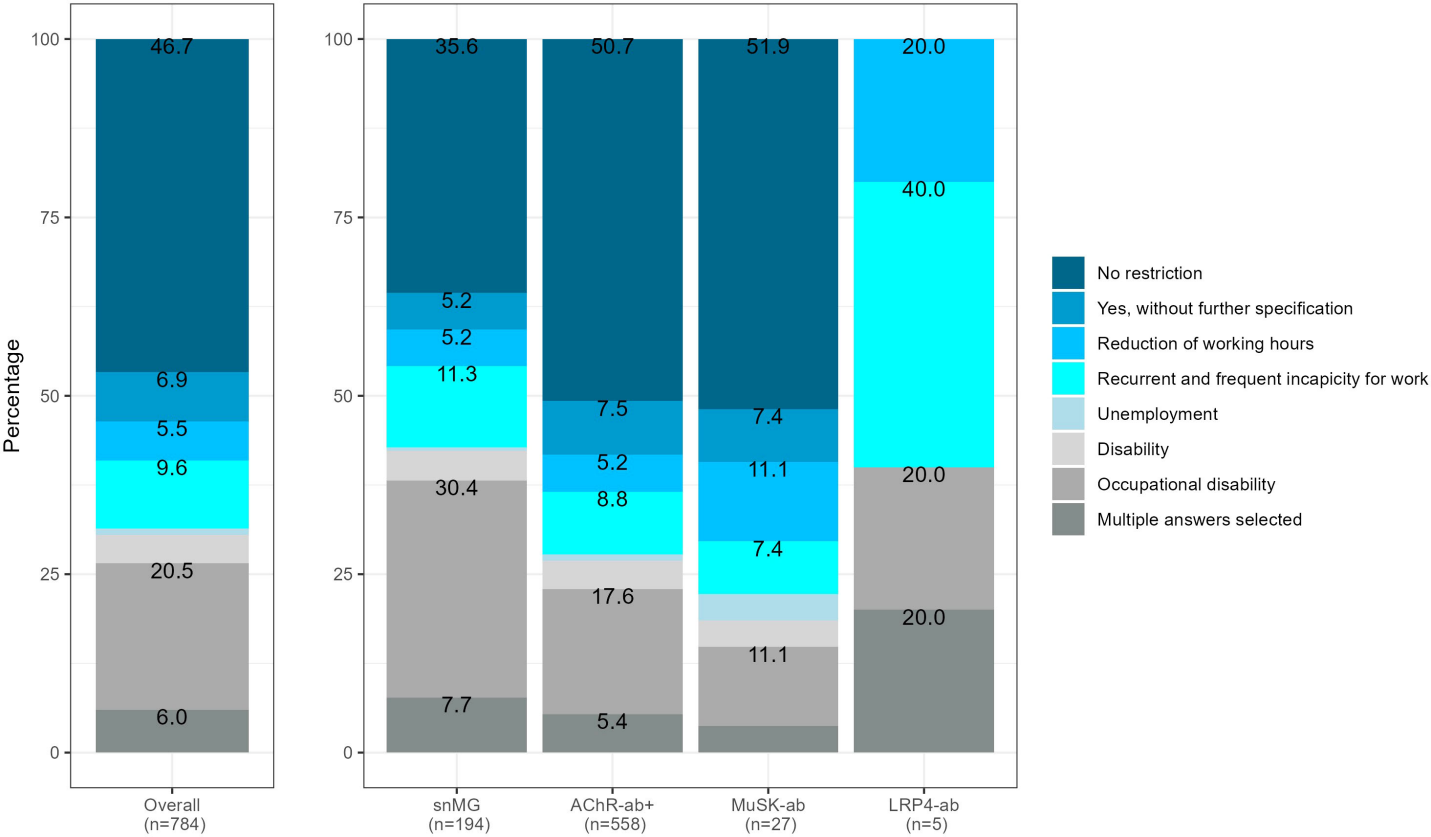
Restriction of employment, referring to 784 patients with available antibody status that were not retired before MG symptom onset, 63 missing, SMD seronegative vs. AChR-ab+ (restriction of employment): 0.39, p-value of the *χ* ^2^ test <0.001.

snMG patients working before MG symptom onset experienced restrictions on employment more frequently compared to AChR-ab+ patients (64.4% vs. 49.3%, SMD 0.39, *p* < 0.001) (**Figure 4**). The most frequent restrictions in snMG patients were occupational disability (30.4% vs. 17.6 in AChR-ab+) and recurrent and frequent incapacity to work (11.3% vs. 8.8% in AChR-ab+) (**Figure 3**). The multivariable logistic regression of restriction for employment (no restriction vs. other categories of **Figure 4**) shows a 1-unit increase in the HADS, increasing the odds of employment restrictions by 10% (95% CI 7%–13%). Even when accounting for HADS, sex, age at disease onset ≥50, and age at symptom onset ≥50, seronegative MG patients have by the factor 1.61 (95% CI 1.07–2.43) increased odds of employment restrictions compared to AChR-ab+ patients (**Supplementary Figure 1**).

Social support was lower in snMG compared to AChR-ab+ MG patients [ESSI-D median 21 (IQR 18/24) vs. 22 (19/25), SMD 0.21, *p* = 0.009) (**Table 4**). There was no difference in antibody constellations regarding the influence of MG on family planning and also no difference in care levels (data not shown in the tables).

### Comparison to the normal population

snMG patients present lower HRQoL measured with the generic SF-36 in a matched-pair comparison to AChR-ab+ patients as well as to the German general population (control). **Figure 3** and **Supplementary Table 1** present mean values of each of the eight domains of the SF-36: mean values of the domains *physical functioning* (SMD 0.31), *physical role functioning* (0.34), *vitality* (0.29), *social role functioning* (0.34), and *pain* (0.33) were lower in snMG compared to AChR-ab+ patients. When comparing snMG with the general population, the differences in quality of life were even more pronounced with significant effects observed in the following domains: *physical functioning* (SMD 1.15), *physical role functioning* (1.2), *vitality* (1.07), *social role functioning* (0.93), and *general health perception* (1.22).

## Discussion

This questionnaire-based cross-sectional study aimed to assess the overall disease burden of MG stratified by autoantibody status by collecting data on sociodemographics, disease severity, treatment, and social impact. Patients with snMG were younger at symptom onset and predominantly women and experienced longer diagnostic delays compared to AChR-ab+ patients. While medication use did not statistically significantly differ, snMG patients reported worse clinical outcomes and a lower response to therapy, including higher disease severity, reduced quality of life, and higher fatigue prevalence and severity. Additionally, snMG patients faced more employment restrictions and demonstrated lower HRQoL across all eight SF-36 domains compared to both AChR-ab+ patients and the general German population in a matched-pair comparison. Our findings highlight the significant medical need for improved diagnostic and therapeutic management in this patient subgroup.

Our study demonstrates that the antibody profile of MG patients significantly impacts the activities of daily living, alongside other factors such as gender, age, and the presence of depression. Patients with snMG had worse MG-ADL scores, indicating greater daily functional impairment. This is also reflected in the overall quality of life of snMG patients, which was notably worse in our cohort compared to AChR-ab+ patients. A Japanese study showed poorer quality of life (MG-QoL15) in snMG patients, but the trend was not as pronounced as in our study (2). By matching snMG patients with AChR-ab+ MG patients in our study, we were able to demonstrate differences across various subdomains of generic HRQoL. This approach enables a direct comparison with the normal population which shows the discrepancy in quality of life.

snMG is recognized as one of the big challenges in the field of MG (9). In the light of a growing trend toward expanded and more precise laboratory diagnostics, clinicians face an increasing diagnostic uncertainty in the absence of autoantibodies which can lead to delay in diagnosis. Diagnostic delay in rare diseases poses a psychological burden (31), and it has been shown to be associated with higher anxiety and depression levels in MG patients (32). Similarly, our study shows higher anxiety and depression levels in snMG patients although this can be multifactorial in nature and might not be solely attributable to the diagnostic delay. Apart from the psychological burden, diagnostic delay likely also affects MG prognosis. Delay in diagnosis inherently leads to delay in therapy. A systematic review demonstrated that early MG diagnosis within the first year is associated with a higher likelihood of achieving clinical remission (33). Previous studies showed that snMG patients are less likely to achieve minimal manifestation status, are less likely to improve on the Myasthenia Gravis Foundation of America (MGFA) classification, and more often do not meet the Patient-Acceptable Symptom State (PASS) criteria (2, 34). This could reflect a hesitancy to escalate treatment in the absence of positive autoantibody testing, potentially due to diagnostic uncertainty. Alternatively, it may indicate the heterogeneity of snMG populations, which could include not only true myasthenic syndromes but also conditions with overlapping conditions or atypical presentations. Our findings with higher MG-ADL score and less likelihood of achieving complete stable remission in snMG patients are consistent with these results, indicating that snMG symptoms are more challenging to manage with conventional treatments. Furthermore, our findings with higher fatigue prevalence in snMG patients are in line with a recent study that showed higher fatigue levels in snMG patients compared to AChR-ab+ patients and also found diagnosis within the first year after symptom onset to be the only protective factor against fatigue (35). In our data, we see that depression and anxiety correlate with the prevalence of fatigue and lower quality of life, which is in line with other studies (35, 36). There is also an effect of psychological wellbeing on employment restrictions, but the antibody status (snMG vs. AChR-ab+) still has a valuable effect and should be taken into account in clinical practice. Our study provides new insights into the social support experiences of patients with snMG, showing that they report lower social support compared to patients with AChR-ab+ MG. While we cannot draw definite conclusions about the underlying causes for this finding, potential contributing factors may include higher disease severity, increased fatigue, and higher prevalence of depressive symptoms. Similar associations have been observed in multiple sclerosis (MS), where lower social support has been linked to a more progressive disease course and poorer quality of life (37–39). Additionally, reduced workforce participation, as indicated by our findings, may also limit social interactions and support.

Limited therapeutic response in snMG patients is further aggravated by limited approvals of novel treatment options in this patient subgroup. Targeted complement inhibition is approved only for AChR-ab+ MG. FcRn inhibition is generally approved only for AChR-ab+ MG (Europe and USA) and only in Japan for snMG. FDA and EMA approval for efgartigimod is restricted to AChR-ab+ MG based on the high placebo responder rate in the ADAPT trial in AChR-ab-negative MG patients (40). Recently, a novel diagnostic paradigm was proposed by international experts with the aim to refine patient selection in clinical trials and to reduce the rate of false-positive snMG diagnosis (9). While stricter criteria may improve clinical trial selection and outcomes, overly rigid application in clinical routine poses the risk of excluding genuine snMG cases and further delaying treatment. On the other hand, functional disorders, myopathies, or congenital myasthenic syndromes (CMS) might be misdiagnosed as snMG, and unnecessary immunotherapy in these patients should be avoided (41–43). In selected cases with inconclusive results in electrophysiological and pharmacological testing, muscle biopsies with assessment of complement and IgG deposition at the neuromuscular junction might be feasible to ascertain MG diagnosis (44, 45).

Direct and indirect healthcare costs in MG patients are high and have been shown to increase with disease severity (46, 47). Our study assessed employment status as an indirect healthcare cost driver. Previous studies on the socioeconomic burden of MG did not include stratification by antibody status (46, 48). Rates of occupational disability in AChR-ab+ MG patients in our study are in line with other studies (47, 49). However, snMG patients in our study reported substantially higher rates of occupational disability, which further highlights the individual and societal burden of snMG.

### Study limitations

Our study, with 1,660 patients, is the largest on this topic to date, and gender distribution aligns with other studies. However, the DMG population may not fully represent the average German MG patient, as it is skewed toward older patients and may overrepresent more severely affected patients. In addition, the patient organization does not check the diagnosis of its members but relies on the self-declaration of being an MG patient. Selection bias cannot be excluded, as more motivated or less sick patients may have responded. To mitigate this effect, we provided a 4-month response window. Recall bias may affect data on past events like symptom onset, though most questions addressed the current situation. Nevertheless, references to the current situation do not always suffice to prevent information bias, as evidenced by four cases reporting implausibly high methotrexate doses, which were subsequently excluded from the analysis. Additionally, while we assessed time to diagnosis in years, we did not capture data in months, which may limit precision. Although our study did not include “diverse” as a gender option, there were no missing data for gender. Antibody status, including seronegativity, was self-reported, raising uncertainty about prior testing and potential false negatives or false positives due to recall bias. To mitigate this limitation, we included the response option “I don’t know” alongside AChR, MuSK, LRP, and seronegative categories to exclude those unaware of their antibody status. This group differs in several aspects, such as age and gender, from the seronegative group, supporting the assumption that most seronegative patients are truly seronegative. Antibody detection rates can vary due to factors such as geography, patient cohort selection, and assay methods (1, 2). Since our study relied on self-reported seronegativity, there is potential uncertainty in classification. The anonymous questionnaire limited the ability to validate responses against clinical data to verify patient’s self-assessment similar to data from the American MGFA registry, which also rely on patient self-reporting. These data are widely regarded as a valuable and substantial contribution to the MG community (50–52). Despite these limitations, our study’s strengths include a large, representative cohort and comprehensive data.

## Conclusion

In conclusion, our study highlights the significant challenges faced by snMG patients. Patients with snMG experience longer diagnostic delays compared to AChR-ab+ patients, report higher disease severity as well as greater fatigue, and face more occupational limitations. Given the high burden of disease in this patient group, it is imperative to improve diagnostic strategies and incorporate snMG patients into clinical trials. Future research should aim to identify potential undetected autoantibodies and develop tailored treatment approaches to better address the needs of this neglected population.

## Data Availability

The raw data supporting the conclusions of this article will be made available by the authors, without undue reservation.
