# Supplementary material for: The burden of disease in seronegative myasthenia gravis: a patient-centered perspective
Source: Front Immunol. 2025 Apr 8;16:1555075. doi: 10.3389/fimmu.2025.1555075 (PMC12011775; doi:10.3389/fimmu.2025.1555075)
Supplement: Supplementary file 1 [file Table1.docx]

Supplement 1 **SF-36 score**: Mean (SD) in numbers corresponding to figure 5.

| **SF-36 Subdomains**  Mean (SD) | **Seronegative**  n=236 | **AChR-ab+**  n=472 | **Control**  n=472 |
| --- | --- | --- | --- |
| Physical functioning | 52.5 (30.0) | 61.8 (29.9) | 82 (22.9) |
| Physical role functioning | 39.2 (41.6) | 53.6 (43.3) | 77.4 (25.6) |
| Vitality | 42.6 (20.2) | 48.8 (21.5) | 61.7 (16.5) |
| Social functioning | 68.4 (42.8) | 70.8 (40.9) | 84.3 (21.8) |
| Social role functioning | 61.6 (29.0) | 71.0 (27.4) | 84.6 (22.2) |
| Emotional well-being | 67.2 (20.0) | 69.8 (18.2) | 73.4 (15.8) |
| Pain | 60.0 (28.2) | 68.5 (25.0) | 69.6 (27.0) |
| General health perception | 45.8 (21.5) | 50.2 (21.8) | 69.1 (17.7) |
