# Supplementary material for: The burden of disease in seronegative myasthenia gravis: a patient-centered perspective
Source: Front Immunol. 2025 Apr 8;16:1555075. doi: 10.3389/fimmu.2025.1555075 (PMC12011775; doi:10.3389/fimmu.2025.1555075)
Supplement: Supplementary file 2 [file Table2.docx]

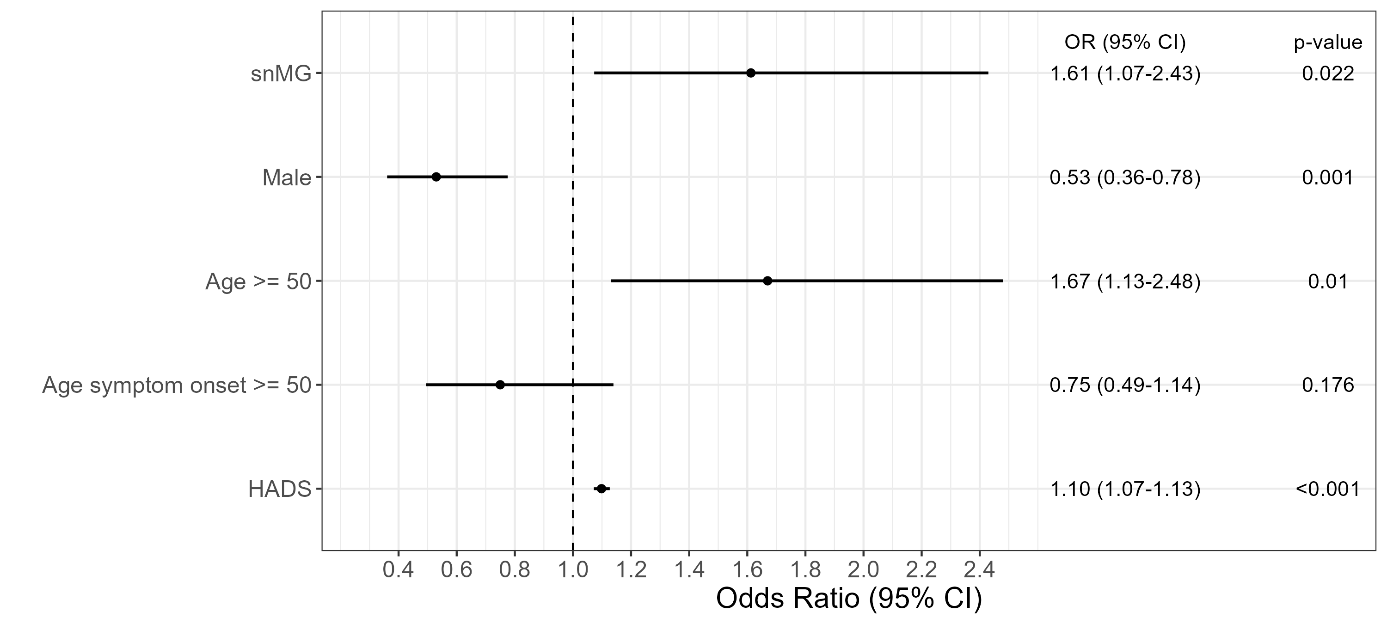


Supplement 2 **Multivariable logistic regression of restrictions for employment.** Complete case analysis including 677 snMG and AchR-ab+ patients (75 missings). OR = odds ratio, CI=confidence interval
